# Molecular mechanisms of traumatic brain injury: the missing link in management

**DOI:** 10.1186/1749-7922-4-7

**Published:** 2009-02-02

**Authors:** Tonny Veenith, Serena SH Goon, Rowan M Burnstein

**Affiliations:** 1 Department of Anaesthesia and Intensive Care, Cambridge University Hospitals NHS Trust Addenbrooke's Hospital, Hills Road, Cambridge, CB2 0QQ, UK

## Abstract

Head injury is common, sometimes requires intensive care unit admission, and is associated with significant mortality and morbidity. A gap still remains in the understanding of the molecular mechanism of this condition. This review is aimed at providing a general overview of the molecular mechanisms involved in traumatic brain injury to a busy clinician. It will encompass the pathophysiology in traumatic brain injury including apoptosis, the role of molecules and genes, and a brief mention of possible pharmacological therapies.

## Introduction and epidemiology

Our understanding of the molecular mechanisms of traumatic brain injury (TBI) has improved over the last decade, but a gap still exists between these advances and their translation into direct clinical care. About 0.5–1 million patients present to hospitals in the UK with TBI. It is the leading cause of disability in people under 40, and severely disables 150–200 people per million annually [1,2]. In the US, TBI affects 1.4 million people, at an estimated annual cost of $56 billion [3]. Diseases of the nervous system (International Classification of Diseases-revision 9) accounted for 8.4% of the total health and social services net public expenditure for 1992 and 1993 in England [4]. The purpose of this review is to look at genetic and molecular influences after an acute head injury and the long term outcome.

Although our ability to assess and predict neurological outcome following TBI has improved, most of the prognostic tools are still poorly validated and therefore rarely used [5]. Understanding the molecular mechanisms and integrating these into clinical practice will help us to predict outcomes more accurately, and will also pave the way for newer treatment modalities and further research.

Current understanding of the basic molecular mechanisms resulting in neurological damage following TBI has sparked several significant attempts to synthesise drugs (*e.g. *Selfotel) [6]. So far these attempts have universally met with little success clinically, but they have provided some insights for future research [6]. Such research has been hampered by a lack of translation of results from animal models into humans. Despite this it is likely that such work, both in animal models and observational studies in patients with acute TBI will continue to shed light in this important subject.

## Pathophysiology of brain injury

Acute TBI is characterised by two injury phases, primary and secondary. The primary brain injury is the direct injury to the brain cells incurred at the time of the initial impact. This results in a series of, biochemical processes which then result in secondary brain injury. The primary aim for the acute management of TBI is to limit the secondary injury. The secondary brain injury is caused by a dynamic interplay between ischaemic, inflammatory and cytotoxic processes. Studies with microdialysis techniques have shown that one of the most significant factors causing secondary brain injury is the excessive release of excitotoxins such as glutamate and aspartate that occurs at the time of the primary brain injury. These excitotoxins act on the N-methyl-D-aspartate channel, altering cell wall permeability with an increase in intracellular calcium and sodium and activation of calcineurin and calmodulin. This ultimately, leads to destruction of the axon [7,8]. Potassium is also released from the cells and absorbed by the astrocytes, in an attempt to restrict the ionic imbalance causing swelling of the cells and ultimately cell death.

There is a complex cascade of cellular inflammatory response following TBI which propagates secondary brain damage. This inflammatory process lasts from hours to days contributing continuously to secondary brain damage. The inflammatory response resulting from an acute TBI is not limited to the brain and multiple organ dysfunction syndromes are commonly seen. The major molecules in the brain involved in this cascade are growth factors, catecholamines, neurokinins, cytokines and chemokines [9].

Interleukins (IL) are proinflammatory cytokines, the levels of interleukins seen in intracerebral bleeds, and clinical signs of inflammation at admission, have correlated well with the magnitude of perilesional oedema and mortality [10,11]. There is a rise in IL-6 and 10 in children following a TBI. The increased level of IL-10 was directly related to mortality in TBI [12]. The rise in inflammatory cytokines (e.g. IL-6) following TBI is a double edged sword; both neurotoxicity and neuroprotection may be induced by it. Inflammatory cytokines facilitate neurotoxicity by encouraging excitotoxicity and the inflammatory response, but simultaneously they facilitate the neurotrophic mechanisms and induction of cell growth factors which are neuroprotective [13]. It has also been shown by Vuylsteke et al that there is an increased gradient of inflammatory marker IL-8 in the brain after cardiopulmonary bypass, which is attenuated by hypothermia [14]. This gradient continued into the postoperative period.

The primary insult also results in an immediate disturbance of the cerebral circulation, resulting in cerebral ischaemia and which contributes significantly to about 90% of deaths after closed head injuries. [15]. Ischaemic brain damage is perpetuated by factors such as hypotension, hypoxia, raised intracranial pressure, oedema, focal tissue compression, damage to microvasculature, and in late phases, vasospasm in the remaining vessels [16,17]. The time sequence after TBI can be arbitrarily divided into an early (phase 1, immediate, with hypoperfusion), intermediate (phase 2, on days 1–3, when hyperaemia can be seen) and a late vasospastic phase (phase 3, on days 4–15, with a marked reduction in blood flow) [17]. These different phases are associated with marked regional variations in cerebral blood flow, with a reduction in blood flow to the surrounding of the ischaemic core, which does not respond to augmentation of cerebral perfusion pressure [18].

## Surviving apoptosis

Programmed cell death (which is often referred to as apoptosis although strictly speaking this refers to the distinct morphological changes after programmed cell death) is a genetic mechanism by which cells are eliminated during development, and is the physiological mechanism by which cells are normally removed in the adult animal [19]. This involves specific genes and proteins which were first described in neuronal development of the round worm [20]. Following TBI there is increased expression of two main sets of genes which are genes encoding for the caspase family of cysteine proteases [including interleukin-1β converting enzyme (ICE) and cpp32] and a family of genes that are homologous to the oncogene Bcl-2 that either promote or suppress cell death. The Bcl-2 gene family controls both caspase dependent and independent apoptosis. [19,21-23]. The endpoint of all these steps is fragmentation of cellular DNA with collapse of the nuclear structure, followed by the formation of membrane-wrapped apoptotic bodies, cleared by macrophages [24].

Apoptosis is now recognised as an important factor in secondary brain injury [25]. Following TBI, two different types of cells are visible; type 1 and 2 cells. The type 1 cells show a classic necrotic pattern (this follows the primary brain injury) and type 2 cells shows a classic apoptotic pattern on microscopy [25,19]. Cells undergoing apoptosis die without membrane rupture and therefore elicit less inflammatory reactions. This is in contrast to the cells undergoing necrosis [26]. There is therefore a suggestion that neuronal apoptosis after TBI may be a protective response by the brain in order to remove injured tissue cells whilst having little effect on remaining brain tissue [27]. Apoptotic cells have been identified within contusions in the acute post-traumatic period, and in regions remote from the site of injury days and weeks after trauma.

Pharmacological strategies to reduce apoptotic cell death have been investigated, [28] For example, rats treated with the caspase-3 inhibitor *N*-benzyloxycarbonyl-Asp-Glu-Val-Asp-fluoromethylketone (DEVD) demonstrate a 30% reduction in lesion volume measured 3 weeks after TBI when compared with vehicle-treated controls [19].

## Long term pathophysiology

Recent advances in the management of severe acute TBI has resulted in improved outcomes for patients who might previously have had poor outcomes. In particular the management of such patients in specialist units has had a significant impact, although the definitive factors contributing to improved outcomes remain elusive. [29]. In recent years there has been increasing interest in elucidating the long term problems experienced by patients following TBI. Further, there have been reports of people developing dementia-like symptoms following relatively minor head injuries (Brain injury with a GCS greater than 13 and without loss of consciousness, as well as an increased incidence of post traumatic stress disorders and depression [30]. TBI causes a generalised atrophy of brain which is proportional to the severity of the injury. [31]. The mechanisms for this are yet to be fully determined. In rats it has been shown that there are multiple antibodies to the amyloid precursor protein and amyloid precursor protein-like proteins for up to six months, which predisposes them to degeneration of the striatum and corpus callosum. This degeneration then leads to progressive brain atrophy and calcifications [32]. In moderate to severe TBI there is a high incidence of hippocampal atrophy which predisposes patients to cognitive decline. When anoxic brain damage was compared to TBI there was no overwhelming evidence of localised nerve damage. This supports the theory that the final mechanism for neurological injury is the same irrespective of the type of initial insult [33].

## Surviving the ischaemic insult: the role of genes

Surprisingly humans are made up of only 20,000 – 25,000 protein-coding genes, and these genes have profound implications on our survival [34]. The genetic constituents not only modify the risk of development of disease and its severity, but also the ability of an organ to repair, heal and function after an injury. In head injured patients the outcomes are variable and cannot easily be predicted. This variability cannot be fully explained by clinical features or by the character of the injury [35]. One of the mechanisms which could explain this is genetic polymorphism. This may also contribute to variability in outcome in the acute response, and functional recovery. A greater understanding of the genetics could aid in the prediction of outcomes and could be targeted for treatment strategies.

Studies in animals using cDNA microarray hybridization technique have shown differential regulation of 86 genes (seven classes) which take part in the physiological and pathological response to TBI. The key classes they encompass include transcription factors, signal transduction genes and inflammatory proteins [36]. Such changes in gene expression are interlinked with both disease processes (for example IL-6 and haemoxygenase-1), and outcome in TBI.

### Genes regulating the inflammatory process

Genetic polymorphisms which involve interleukin-6 (IL-6) and haemoxygenase -1 (HO-1) may influence the inflammatory effects seen after TBI [37]. There are two genetic polymorphisms associated with increased IL-6 levels in blood -174G>C and -572G>C, the presence of which not only increased the risk of development of coronary and cerebral aneurysms but also increased the mortality when they ruptured [38]. Haemoxygenase is a rate-limiting enzyme in haem catabolism and the inducible form of haemoxygenase is haemoxygenase-1 (HO-1). There is an increased expression of HO-1 in the injured rat brain model. The end product molecules influence tissue redox homeostasis under a wide range of pathophysiological conditions including TBI [38].

### Genes regulating the vascular responses

Cerebral ischaemia results in an activation of the hypoxia-inducible factor-1 and 2 (HIF 1&2) genes. HIF-1 activates the transcription of numerous genes including vascular endothelial growth factor (VEGF), glucose transporter-1 (Glut1), Epo, transferrin (Tf), and the transferrin receptor (TfR) all of which have been shown to be neuroprotective in animal models after TBI [39]. Vascular endothelial growth factor (VEGF) is the main regulator of angiogenesis, and in the normal adult brain and is predominantly expressed in the epithelial cells of the choroid plexus, astrocytes and neurons (such as granule cells of the cerebellum) [40]. Following cerebral ischaemia there is upregulation of both VEGFR-2 and VEGF expression. [41]. Somewhat confusingly HIF-1 upregulation and increased VEGF expression have been associated with the development of cerebral oedema and neuronal death following brain injury [Chen et al, 2008, Neurobiology of Disease] whilst also being implicated in peri infarct neuroprotection [42] Deficiencies of HIF genes in mice are associated with embryonic death due to cardiac, vascular, and neural malformations [43].

### Genes regulating the neuronal response to TBI

Apolipoprotein epsilon (APOE) is a multifunctional protein involved predominantly in the transport of cholesterol, maintenance of microtubules, neurones, and neural transmission. This gene is important in the neuronal response of the brain to injury and in the subsequent repair processes. There are three different variants (ε2, ε3, and ε4) to this gene and the variant ε4 situated on chromosome 19 is associated with the development of Alzheimer's disease, and predisposes to worse outcome in TBI [44-46].

The presence APOE-ε4 is associated with a poor outcome in cognitive dysfunction and functionality following brain injury rehabilitation [47-49]. It is also associated with a rapid cognitive decline in Alzheimer's disease [50] and in autopsy studies has been demonstrated to incur a significantly increased risk of development of cerebral amyloid angiopathy [51]. In larger retrospective studies of outcome following TBI, the presence of APOE-ε4 correlates with a significantly worse outcome in young patiens (aged 0–15 years). This correlation reduces with age, with, neutralisation at 55 years [45].

The P53 gene is important in the regulation of apoptosis; this gene exhibits a common polymorphism that results in either proline or arginine at amino acid 72. Arg/Arg genotype of the Arg72Pro polymorphism in p53 is associated with an increased likelihood of a poor outcome at discharge from the surgical intensive care unit following TBI. [52]

### Genes regulating the catecholamines

There are three isoforms of the enzyme catechol-o-methyltransferase (COMT) encoded by 3 genetic polymorphisms (COMT Val/Val, COMT Val/Met, and COMT Met/Met). This enzyme is associated with inactivation of dopamine and norepinephrine and is thought to functionally modulate dopamine neurons, thus influencing frontal-executive functioning. In a study by Lipsky et al (2005) in patients with TBI, polymorphism (Val/Val), and presumably lower cortical DA levels, resulted in worse performance on the Wisconsin Card Sorting Test compared to patients with the low activity polymorphism (Met/Met) and presumably higher cortical DA levels [53].

## Pharmacological therapies

A variety of pharmacological agents have been trialed, all of which have shown promising results in animal models, but when translated into the clinical setting have universally failed to influence outcome following TBI. These agents include Selfotel, Cerestat, CP 101–606, D-CPP-ene, Steroids, tirilazad, PEG-SOD, IGF-1/growth hormone, Nimodipine, Bradycor, Dexanabinol, SNX-III, and anticonvulsants (such as Valproate and Magnesium Sulphate). The neuroprotective actions of these agents result from a variety of mechanisms of action, including antagonism of glutamate (Selfotel and CP 101–606), and free radical scavenging (PEG-SOD) [6].

Dexanabinol is a synthetic chemical analogue of the active component of marijuana. It is a non-competitive inhibitor of the NMDA receptor, a free radical scavenger and antioxidant, and an inhibitor of the pro-inflammatory cytokine TNF alpha [6].

Steroids are used with good effect in the treatment of brain oedema associated with brain tumours, and have been shown in laboratory studies to reduce free radical production and have a protective effect on the brain. However, several clinical studies in TBI have shown no clear beneficial effect on outcome or intracranial pressure [6].

### Catecholamines

One of the key factors in the management of TBI is maintenance of cerebral perfusion pressure and cerebral blood flow, and systemic administration of catecholamines is often used to achieve this. Circulating endogenous catecholamines are increased in TBI due to stimulation of the sympatho-adrenal axis. Endogenous circulating catecholamines are a readily quantifiable marker that predicts the outcome in TBI [52,54]. It has been shown in rodents that optimal synthesis of catecholamines in the brain is critical to a working memory. TBI results in activation of tyrosine hydroxylase (TH) in the brain. This is the rate limiting step in catecholamine synthesis and changes in activation of TH result in altered catecholamine signalling in the prefrontal cortex which impacts on memory [55].

### Neurotrophins

Neurotrophins are normally found in cell bodies and the projections of neurons, and they facilitate neuronal survival and differentiation [56,57]. They include nerve growth factor (NGF), brain-derived neurotrophic factor (BDNF), neurotrophin-3 (NT-3), neurotrophin-4 (NT-4) and neurotrophin-5 (NT-5). Of the neurotrophic agents, BDNF shows the most promise in the future management of brain injury. Animals treated with BDNF following TBI, showed an improvement in cognitive function and regeneration of the neural network which resembled developmental neuroplasticity. This was directly related to improvement in synchronized movement and spatial orientation [58,59]. Unfortunately there is no convincing evidence for the use of these drugs in humans [60].

## Conclusion

This review emphasises that the molecular mechanisms underlying secondary brain damage following TBI are complex. Our understanding of these mechanisms has increased significantly in recent years, but is far from complete. Advances in the acute management of TBI, is likely to be dependant both on an improved understanding of these mechanisms, as well as the translation of such knowledge into the development of new molecules and techniques to improve the clinical outcome.

## Competing interests

The authors declare that they have no competing interests.

## Authors' contributions

TV researched the topic and wrote the draft article, and together with SG structured the article. RB is the supervisor for this article. All authors read and approved the final manuscript.
